# The relationship between glucocerebrosidase mutations and Parkinson disease

**DOI:** 10.1111/jnc.13385

**Published:** 2016-02-10

**Authors:** Anna Migdalska‐Richards, Anthony H. V. Schapira

**Affiliations:** ^1^ Department of Clinical NeurosciencesUCL Institute of NeurologyLondonUK

**Keywords:** alpha‐synuclein, Gaucher disease, glucocerebrosidase 1 (*GBA1*), lysosome, mitochondria, Parkinson disease

## Abstract

Parkinson disease (PD) is the second most common neurodegenerative disorder after Alzheimer disease, whereas Gaucher disease (GD) is the most frequent lysosomal storage disorder caused by homozygous mutations in the glucocerebrosidase (*GBA1*) gene. Increased risk of developing PD has been observed in both GD patients and carriers. It has been estimated that *GBA1* mutations confer a 20‐ to 30‐fold increased risk for the development of PD, and that at least 7–10% of PD patients have a *GBA1* mutation. To date, mutations in the *GBA1* gene constitute numerically the most important risk factor for PD. The type of PD associated with *GBA1* mutations (PD‐*GBA1*) is almost identical to idiopathic PD, except for a slightly younger age of onset and a tendency to more cognitive impairment. Importantly, the pathology of PD‐*GBA1* is identical to idiopathic PD, with nigral dopamine cell loss, Lewy bodies, and neurites containing alpha‐synuclein. The mechanism by which *GBA1* mutations increase the risk for PD is still unknown. However, given that clinical manifestation and pathological findings in PD‐*GBA1* patients are almost identical to those in idiopathic PD individuals, it is likely that, as in idiopathic PD, alpha‐synuclein accumulation, mitochondrial dysfunction, autophagic impairment, oxidative and endoplasmic reticulum stress may contribute to the development and progression of PD‐*GBA1*. Here, we review the *GBA1* gene, its role in GD, and its link with PD.

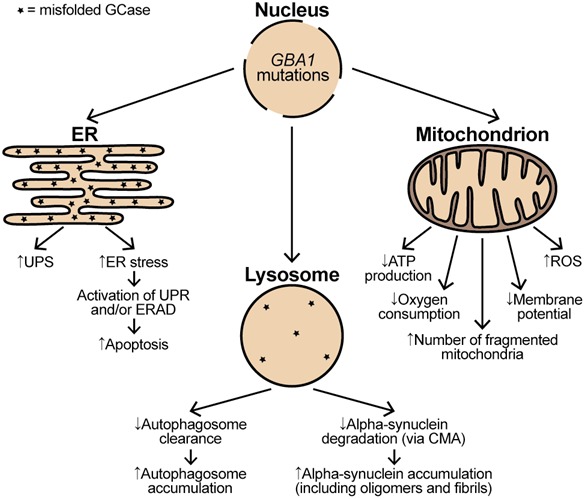

The impact of glucocerebrosidase 1 (*GBA1*) mutations on functioning of endoplasmic reticulum (ER), lysosomes, and mitochondria. *GBA1* mutations resulting in production of misfolded glucocerebrosidase (GCase) significantly affect the ER functioning. Misfolded GCase trapped in the ER leads to both an increase in the ubiquitin–proteasome system (UPS) and the ER stress. The presence of ER stress triggers the unfolded protein response (UPR) and/or endoplasmic reticulum‐associated degradation (ERAD). The prolonged activation of UPR and ERAD subsequently leads to increased apoptosis. The presence of misfolded GCase in the lysosomes together with a reduction in wild‐type GCase levels lead to a retardation of alpha‐synuclein degradation via chaperone‐mediated autophagy (CMA), which subsequently results in alpha‐synuclein accumulation and aggregation. Impaired lysosomal functioning also causes a decrease in the clearance of autophagosomes, and so their accumulation. *GBA1* mutations perturb normal mitochondria functioning by increasing generation of free radical species (ROS) and decreasing adenosine triphosphate (ATP) production, oxygen consumption, and membrane potential. *GBA1* mutations also lead to accumulation of dysfunctional and fragmented mitochondria.

**This article is part of a** special issue on Parkinson disease **.**

**Abbreviations used**
Alu: *Arthrobacter luteus*
ATG: methionine
BiP: binding immunoglobulin protein
CBE: condruitol β‐epoxide
CHOP: C/EBP homologous protein
CLEAR: coordinated lysosomal expression and regulation
CMA: chaperone‐mediated autophagy
CNS: central nervous system
DJ‐1: Parkinson protein 7
ER: endoplasmic reticulum
ERAD: ER‐associated degradation
FDA: Food and Drug Administration
GD: Gaucher disease
*GBA1*: glucocerebrosidase
GCase: glucocerebrosidase enzyme
LIMP2: lysosomal membrane protein 2
LRRK2: leucine‐rich repeat kinase 2
MPR: mannose‐6‐phosphate receptor
mRNA: messenger RNA
PARK2: parkin RBR E3 ubiquitin protein ligase
PCR: polymerase chain reaction
PC12: pheochromocytoma
PD: Parkinson disease
PD‐*GBA1*: Parkinson‐*GBA1*
pH: power of hydrogen
PINK1: PTEN‐induced putative kinase 1
PI4K: phosphatidylinositol 4‐kinase
PTEN: phosphatase and tensin homolog
SNCA: alpha‐synuclein
TFEB: transcription factor EB
UPR: unfolded protein response

## Glucocerebrosidase 1 gene

The human glucocerebrosidase 1 (*GBA1)* gene maps to the 1q21 region and consists of 11 exons within a 7.6 kb sequence (Horowitz *et al*. 1989; Cormand *et al*. 1997). Approximately, 16 kb downstream of *GBA1* lies an almost identical sequence consisting of 11 exons within a 5.7 kb sequence (Horowitz *et al*. 1989; Sorge *et al*. 1990). Despite large deletions of *Arthrobacter luteus* sequences flanked by direct repeats in the introns, this pseudogene shares 96% coding sequence similarity with the functional gene (Horowitz *et al*. 1989). As some mutations found in the functional gene can naturally occur within the pseudogene, the presence of the highly homologous pseudogene in close proximity to the functional gene complicates molecular identification of known and novel *GBA1* mutations. Therefore, a method allowing the *GBA1* gene to be distinguished from the pseudogene is crucial for reliable molecular diagnosis. One such method utilizes a long‐template polymerase chain reaction (PCR) where genomic fragments of differing lengths from both the gene and the pseudogene are amplified simultaneously, before being purified and subsequently used for mutation identification (Tayebi *et al*. 1996).

Northern blot analysis revealed the existence of at least two *GBA1* mRNAs (2.2 and 2.6 kb in length) that arise as a result of alternate polyadenylation sites (Horowitz *et al*. 1989).The *GBA1* mRNA has two in‐frame methionine start codons located in exons 1 and 2, and both methionines are translated to produce functional protein *in vitro* (Sorge *et al*. 1985, 1987a). The protein using the start codon in exon 1 contains a 39‐amino acid signal peptide, while the protein arising from the start codon in exon 2 contains only a 19‐amino acid signal peptide. Both are processed to a 496‐amino acid long mature protein after their signal peptide sequences are removed, which is a common occurrence for secreted proteins like glucocerebrosidase (Sorge *et al*. 1987b).

*GBA1* encodes glucocerebrosidase (GCase), a lysosomal enzyme that catalyses the hydrolysis of glycolipid glucocerebroside to ceramide and glucose (Beutler 1992). Glucocerebrosidase is ubiquitously expressed in all types of tissues (The Human Protein Atlas). As with other lysosomal proteins, GCase is synthetized in the rough endoplasmic reticulum (ER). However, in contrast to the majority of other lysosomal proteins, transport of GCase from the ER to lysosomes is not mediated by mannose‐6‐phosphate receptors, but via lysosomal membrane protein 2 (LIMP2). GCase binds to a coiled‐coil domain in the lumenal region of LIMP2 at neutral pH of the ER, and both proteins persist together through the Golgi apparatus and endosomes into the lysosome, where acidic pH facilitates their dissociation (Reczek *et al*. 2007). Sequential traversing of GCase and the LIMP2 complex is dependent on two phosphatidylinositol 4‐kinases (PI4Ks), with the catalytic activity of the PI4K type IIIβ (PI4KIIIβ) kinase required for exit of the complex from the Golgi apparatus, and the PI4K type IIα (PI4KIIα) kinase needed for correct sorting of the complex from endosomes to lysosomes (Jovic *et al*. 2012).

The crystal structure of GCase, first obtained in 2003, revealed that GCase has three non‐continuous domains. Domain I consists of a three‐stranded anti‐parallel β‐sheet. It contains two disulphide bridges, which may be involved in GCase folding. Domain II consists of two β‐sheets that form an immunoglobulin‐like domain. Finally, domain III consists of an eight‐stranded β/α triose phosphate isomerase (TIM) barrel and contains the catalytic site of GCase (Dvir *et al*. 2003).

The human GCase is glycosylated at four out of five available asparagine residues (N19, N59, N146, and N270, but not N462), and glycosylation is required for its catalytic function. It has been shown that mutations of asparagine residues N59, N146, and N270 do not affect GCase catalytic function and GCase interaction with active site‐directed inhibitors and activators. However, mutations of asparagine residue N19 result in production of catalytically inactive enzyme (Berg‐Fussman *et al*. 1993).

## Gaucher disease

Gaucher disease (GD) is named after Dr Philippe Gaucher who, in 1882, first described a young woman with an enlarged spleen containing unusual looking cells (Gaucher 1882). GD is the most common autosomal recessive lysosomal storage disorder. Its most characteristic hallmark is the presence of glucocerebroside‐laden macrophages in the liver, spleen, and bone marrow, known as Gaucher cells. The most common clinical features of GD include hepatosplenomegaly, thrombocytopenia, anemia, bone involvement with osteopenia, osteoporosis, and bone pain because of bone infarcts or pathological fractures (Beutler and Grabowski 2001). GD has been traditionally divided into three distinct clinical forms based on age of onset and involvement of the central nervous system (CNS) (Cox and Schofield 1997). Type 1 GD (OMIM, #230800) is by far the most common form and is classified as non‐neuronopathic since it does not affect the CNS. The disease course of type 1 GD is very heterogeneous with some patients developing first symptoms in early childhood and others not manifesting any symptoms until well into adulthood. Type 2 and type 3 GD are classified as neuronopathic forms. Type 2 GD (OMIM, #230900) is the acute neuronopathic form with onset in infancy. It is characterized by rapid progression of neurological symptoms leading to death within 2 years of age. Infants are apparently normal for the first few months of life, after which they display hepatosplenomegaly, developmental regression, growth arrest, and rapid neurological decline (Stone *et al*. 2000). Type 3 GD (OMIM, #231000) is a chronic neuronopathic form with onset in early childhood. Unlike type 2, it is characterized by slow progression of neurological symptoms with parallel manifestation of all clinical symptoms diagnosed in type 1 GD leading to death in early adulthood (Beutler and Grabowski 2001). The prevalence of GD in the general population is about 1/40 000–1/50 000 live births, while the incidence of GD among Jews of Ashkenazi origin is up to 1/450 live births with a carrier frequency of about 6% (Grabowski 2008; National Organization of Rare Disorders 2013; Bronstein *et al*. 2014).

GD is caused by mutations in the *GBA1* gene that lead to deficiency of the lysosomal enzyme glucocerebrosidase (GCase). Nearly, 300 pathogenic changes in *GBA1*, including point mutations, splice‐site mutations, deletions, insertions, and recombinant alleles containing genomic sequences of both the gene and pseudogene, have been identified (Hruska *et al*. 2008). These alterations lead to production of misfolded mutant enzymes with significantly reduced activity. GCase activity in GD patients is typically only 10–20% of that in normal individuals, while GCase activity in carriers is about 50%. It is thought that, not only GCase deficiency, but also ER stress triggered by the presence of misfolded GCase contribute to the pathogenesis of GD. The two most common *GBA1* mutations identified in GD patients are N370S and L444P (Sidransky and Lopez 2012). Interestingly, the type of mutation is broadly predictive of GD form, as patients homozygous or compound heterozygous for the N370S mutation exclusively develop type 1 GD, patients homozygous for the L444P mutation are most likely to develop type 3 GD, while patients identified with a complex allele and a heterozygous L444P mutation are most likely to develop type 2 GD (Grabowski 2008; Sidransky 2012). The severity of the GD phenotype in relation to the observed mutation might be explained by the effect, which the particular mutation has on the GCase structure. Namely, the N370S mutation, which is situated on the longest α‐helix of GCase at the interface of domain II and III, does not directly affect the catalytic activity of GCase since it located too far from the catalytic site of domain III. The L444P mutation, which is situated at the hydrophobic core of domain II, causes conformational changes to the core and to domain II, which might result in the generation of unstable GCase (Dvir *et al*. 2003).

Currently, two types of treatment are available for patients with GD: enzyme replacement therapy (using imiglucerase, velaglucerase alfa, or taliglucerase alfa) and substrate reduction therapy (using misglustat) (Bennett and Mohan 2013). Although these treatments greatly improve the peripheral (non‐neuronopathic) features of the disease, they are ineffective for treatment of neuronopathic symptoms of type 2 and type 3 GD as they do not cross the blood–brain barrier (Bennett and Mohan 2013). Emerging treatments in the form of small molecular chaperones designed to cross the blood–brain barrier, which are able to bind misfolded mutant GCase in the endoplasmic reticulum, help its correct folding and subsequently assist its transport to the lysosomes, give great promise for treatment of the neuronopathic features of GD. The ability of small molecular chaperones to bind to misfolded GCase (and subsequently to induce proper folding of mutant GCase that in turn would increase functional GCase levels in the lysosomes) is particularly important, as it has been shown that majority of *GBA1* mutations lead to the production of misfolded GCase (Sawkar *et al*. 2002; Bernier *et al*. 2004; Suzuki *et al*. 2009; Patnaik *et al*. 2012). Several such small molecular chaperones that lead to an increase in GCase activity have been investigated in both cell and animal models (Sawkar *et al*. 2002; Bernier *et al*. 2004; Steet *et al*. 2006; Maegawa *et al*. 2009; Bendikov‐Bar *et al*. 2011; Patnaik *et al*. 2012; Bendikov‐Bar *et al*. 2013; Luan *et al*. 2013). GCase inhibitors (e.g. *N*‐(*n*‐nonyl)deoxynojirimycin, and isofagomine) were the first chaperones shown to increase GCase activity. Both *N*‐(*n*‐nonyl)deoxynojirimycin and isofagomine increased GCase activity by increasing GCase trafficking to the lysosomes through specific, but reversible binding of GCase in the ER. Although they led to an increase in GCase activity in fibroblasts derived from Gaucher patients, it has been demonstrated that their clinical application is compromised because of difficulties in balancing chaperone activity with the direct inhibition of GCase activity (Sawkar *et al*. 2002; Bernier *et al*. 2004; Steet *et al*. 2005). Subsequently, a class of pyrazolopyrimidines, the first non‐inhibitory small molecular chaperones, was discovered. The pyrazolopyrimidines act as GCase activators that lead to both an increase in GCase translocation to the lysosomes and in GCase activity in wild‐type and mutant (N370S/N370S and L444P/L444P) human fibroblasts (Patnaik *et al*. 2012). Another promising small molecular chaperone, ambroxol hydrochloride (from now on commonly referred to as ambroxol), was identified after screening the library of Food and Drug Administration‐approved drugs with a thermal denaturation assay using wild‐type GCase (Maegawa *et al*. 2009). Ambroxol has long been used to treat airway mucus hypersecretion and hyaline membrane disease in newborns, which demonstrates its non‐toxicity to humans. Ambroxol acts as a GCase mixed‐type inhibitor with its inhibitory property toward GCase being highest at the neutral pH of the endoplasmic reticulum and void at the acidic pH of the lysosomes where functional GCase is required (Maegawa *et al*. 2009). Several studies have demonstrated that ambroxol treatment of different lines of cultured human GD fibroblasts results in a significant increase in GCase activity (Maegawa *et al*. 2009; Bendikov‐Bar *et al*. 2011, 2013; Luan *et al*. 2013; McNeill *et al*. 2014). However, whether an increase in GCase activity is because of ambroxol's chaperone activity alone remains to be clarified, as it has been shown in a fibroblast model that ambroxol increased GCase activity by activating the coordinated lysosomal expression and regulation network (coordinated lysosomal expression and regulation) via the action of transcription factor EB, which in turn led to an increase in lysosomal biogenesis. An enhancement of lysosomal mass would most likely lead to an increase in GCase activity, not necessarily via the elevated chaperone activity of ambroxol (McNeill *et al*. 2014). Currently available data from ambroxol‐treated wild‐type and transgenic mice carrying human *GBA1* mutations has convincingly shown increase in GCase activity in the peripheral organs, but variable change in the brain. Hence, more studies are required to determine the effect of ambroxol on GCase activity in the CNS (Luan *et al*. 2013; Sanders *et al*. 2013).

## The link between *GBA1* and Parkinson disease

The link between GD and Parkinson disease (PD) was initially regarded as incidental, thus the first publications describing Gaucher patients with Parkinsonian features originated from individual clinics (McKeran *et al*. 1985; Turpin *et al*. 1988; Tayebi *et al*. 2001). The increasing number of reports suggesting the association between mutations in the *GBA1* gene and PD led to more comprehensive studies focusing on several Gaucher patients with PD (Bembi *et al*. 2003; Tayebi *et al*. 2003; Varkonyi *et al*. 2003). Moreover, an increased proportion of PD cases in GD carriers compared to the general population further indicated the association of the *GBA1* gene with PD (Goker‐Alpan *et al*. 2004; Halperin *et al*. 2006). This finding led researchers to investigate whether the frequency of *GBA1* mutations is increased in PD. The first such study identified *GBA1* mutations in 12 of 57 (21%) PD postmortem brains (Lwin *et al*. 2004). This study not only further supported the link between *GBA1* and PD, but also indicated that both heterozygous and homozygous mutations in *GBA1* might be associated with PD. Subsequently, multiple PD patients were investigated to establish the prevalence of *GBA1* mutations, which highlighted the importance of routine *GBA1* mutation screening when diagnosing PD individuals. The approach taken by different research groups varied, as some screened only for the most common *GBA1* mutations, while others sequenced all exons of the gene (Tables 1, 2, 3). Overall, data acquired from multiple studies showed that the frequency of heterozygous *GBA1* mutations varied between 2.3–9.8% in the European population of non‐Ashkenazi Jewish origin, 16.9–31.3% in the European population of Ashkenazi Jewish origin, 1.8–8.7% in the Asian population, and 2.9–8.0% in the combined North–South American population (Tables 1, 2, 3). The most common mutations identified in the European population of non‐Ashkenazi Jewish origin were L444P and N370S, and both mutations were found with similar frequency (Table 1). Interestingly, in the European population of Ashkenazi Jewish origin, the N370S mutation was the most predominant, while in the Asian and North–South American populations, the L444P mutation was the most prevalent (Tables 1, 2, 3). Moreover, most studies conducted on European PD individuals of Ashkenazi Jewish origin did not detect any L444P mutations, while the majority of studies carried out on PD patients from the Asian population did not identify any N370S mutations (Tables 1 and 2). Nevertheless, given the observed discrepancies in incidence of *GBA1* mutations within the same population, and the limited number of cases and controls available for individual studies, a much bigger study was required to provide a conclusive answer about the frequency of *GBA1* mutations among people of different origin. A multicenter meta‐analysis collected a total of 5691 PD patients and 4898 controls (Sidransky *et al*. 2009). They analyzed 780 PD individuals and 387 controls of Ashkenazi Jewish origin for both the N370S and L444P mutations, and found that 15% of patients and 3% of controls had one of the two mutations. They also screened 4911 PD individuals and 4511 controls of non‐Ashkenazi Jewish origin and found that 3% of patients and less than 1% of controls had at least one of the two mutations. Finally, they sequenced the entire *GBA1* gene in 1883 PD individuals of non‐Ashkenazi Jewish origin and found that 7% of patients carried *GBA1* mutations. To summarize, mutations in the *GBA1* gene constitute numerically the most important predisposing risk factor for developing PD. Both homozygous and heterozygous *GBA1* confer a 20‐ to 30‐fold increased risk for the development of PD, and it is estimated that approximately 5–10% of PD patients have a *GBA1* mutation (Sidransky *et al*. 2009; Bultron *et al*. 2010; McNeill *et al*. 2012a,b). However, not all *GBA1* mutant carriers will develop PD, and it is currently estimated that 30% will develop the disease by age 80 years (Lesage and Brice 2009; Lesage *et al*. 2011).

**Table 1:** Frequency and type of *GBA1* mutations found in the European population

Number		% with mutation		Method	Alterations found	Most common mutation(s)	Origin	Reference
PD cases	Control cases	PD cases	Control cases					
2350	1111	4.5	0.63	Screening of *GBA1* exon 9 and 10	N370S, L444P, D443N, and IVS10+1G>T	–	Italian	Asselta *et al*. 2014
259	–	3.5	–	Sequencing of *GBA1* exons	L444P*,* N370S*,* N462K*,* R463C, and R257Q	N370S (33.33%) L444P (33.33%)	British	Winder‐Rhodes *et al*. 2013
360	348	5.8	1.4	Sequencing of *GBA1* exon 8 to 11	N370S, D409H, H255Q, L444P, A456P, R463C, and RecNciI	N370S (33.33%)	Serbian	Kumar *et al*. 2013
311	474	2.3	1.7	Genotyping for L444P and N370S	L444P and N370S	N370S (57.14%) L444P (42.86%)	Norwegian	Toft *et al*. 2006
225	186	9.8	0.5	Sequencing of *GBA1* exons	M123T, L144V, G202R, I260T, T369M, N370S, W393R, D409H, L444P, RecNciI, and S488T	L444P (27.27%) N370S (22.72%)	Spanish	Setó‐Salvia *et al*. 2012
330	240	2.7	0.4	Genotyping for L444P and N370S	L444P and N370S	L444P (66.67%)	Russian	Emelyanov *et al*. 2012
1391	391	6.7	1.0	Sequencing of *GBA1* exons	Lots of different mutations	N370S (47.40%)	French	Lesage *et al*. 2011
205	206	10.24	3.39	Genotyping for N370S, D409H, L444P, H255Q, R120W, Y108C, IVS6‐2A>G, and IVS10‐1G>A	N370S, L444P, D409H; H255Q, D409H, Y108C, IVS10‐1G>A	N370S (28.57%) L444P (28.57%)	Greek	Moraitou *et al*. 2011
230	430	6.1	0.7	Sequencing of *GBA1* exons	N370S, N396T, D409H, and L444P	N370S (33.33%) N396T (33.33%)	Portuguese	Bras *et al*. 2009
172	132	3.4	0.3	Sequencing of *GBA1* exons	L445P, D409H, E326K, H255Q, R329H, L268L, S271G, T428K, and V460L	H255Q (36.36%) L444P (18.18%)	Greek	Kalinderi *et al*. 2009
790	257	4.18	1.17	Sequencing of *GBA1* exons	L444P, D443N, R463C, RecNciI, RecA456P, N370S, D409H, D380A, c.1263‐1317del55, R257Q, G193E, R131C, K7E, and V458L	L444P (33.33%) N370S (24.24%)	British	Neumann *et al*. 2009
420	4138	17.9	4.2	Genotyping for N370S, R496H, 84GG, IVS2+1, V394L, D409H, L444P, and RecTL	N370S, R496H, 84GG, IVS2+1, V394L, L444P, and RecTL	N370S (61.33%)	Ashkenazi Jewish (Israeli)	Gan‐Or *et al*. 2008
395	483	2.8	0.2	Genotyping for L444P and N370S	L444P and N370S	L444P (72.73%)	Italian	De Marco *et al*. 2008
178	85	16.9	7.1	Sequencing of *GBA1* exons	N370S, R496H, E326K, T369M, P175P, and 84insGG	N370S (78.33%)	Ashkenazi Jewish (American)	Clark *et al*. 2007
99	1543	31.3	6.2	Genotyping for N370S, L444P, 84GG, IVS2+1G>A, V394L, and R496H	N370S and 84GG	N370S (83.87%)	Ashkenazi Jewish (Israeli)	Aharon‐Peretz *et al*. 2004

**Table 2:** Frequency and type of *GBA1* mutations found in the Asian population

Number		% with mutation		Method	Alterations found	Most common mutation(s)	Origin	Reference
PD cases	Control cases	PD cases	Control cases					
184	130	8.7	5.4	Sequencing of *GBA1* exons	c.334_338delCAGAA L264I, L314V, R163Q, F213I, E326K, S364S, F347L, V375L, L444P, RecNciI, and Q497R	L444P (31.25%)	Chinese	Yu *et al*. 2015
480	395	5	0.5	Sequencing of *GBA1* exons	L444P, N386K, P428S, IVS2þ1G>A, IVS9þ3G>C, IVS10‐9_10GT>AG, and c.1309delG	L444P (58%)	Thai	Pulkes *et al*. 2014
195	443	3.08	0.0	Genotyping for L444P, N370S, and R120W mutations	L444P	L444P (100%)	Chinese	Zhang *et al*. 2012
277	291	3.2	0.0	Sequencing of *GBA1* exons	N188S, P201H, R257Q, S271G, and L444P	R257Q (33.33%) L444P (22.22%)	Korean	Choi *et al*. 2012
208	298	3.4	0.3	Genotyping for L444P, N370S, and R120W mutations	L444P	L444P (100%)	Chinese	Wang *et al*. 2012
967	780	3.72	0.26	Sequencing of *GBA1* exons (30 cases) Genotyping for L444P, D409H, R120W, L174P, and Q497R mutations (all individuals)	L444P, RecNciI, and D409H	L444P (75%)	Chinese	Huang *et al*. 2011
328	300	1.8	0.7	Genotyping for N370S mutation	N370S	N370S (100%)	Chinese	Hu *et al*. 2010
616	411	3.2	0.2	Genotyping for L444P mutation	L444P	L444P (100%)	Chinese	Mao *et al*. 2010
402	412	2.74	0.0	Genotyping for L444P, N370S, F213I, and R353W mutations	L444P	L444P (100%)	Chinese	Sun *et al*. 2010a,b
331	347	8	0.0	Genotyping for L444P and N370S mutations	L444P	L444P (100%)	Chinese	Tan *et al*. 2007
518	339	3.1	1.2	Genotyping for L444P, RecNciI, and R120W mutations	L444P, RecNciI and R120W	L444P (81.25%)	Taiwanese	Wu *et al*. 2007
92	92	4.1	1.1	Sequencing of *GBA1* exons	L444P, D409H, L174P, and Q497R	Each mutation (25%)	Taiwanese	Ziegler *et al*. 2007

**Table 3:** Frequency and type of glucocerebrosidase (*GBA1*) mutations found in the combined North–South American population

Number		% with mutation		Method	Alterations found	Most common mutation(s)	Origin	Reference
PD cases	Control cases	PD cases	Control cases					
128	252	5.47	0.0	Genotyping for L444P and N370S mutations	L444P	L444P (100%)	Mexican	González‐Del Rincón Mde *et al*. 2013
65	267	3.08	0.0	Genotyping for N370S, L444P and G377S	L444P	L444P (100%)	Brazilian	Spitz *et al*. 2008
721	554	2.9	0.4	Genotyping for L444P and N370S mutations	L444P and N370S	N370S (52.38%) L444P (47.62%)	American	Mata *et al*. 2008
100	94	8	2.1	Sequencing of *GBA1* exons	N370S, T369M, D409H, and RecNciI	L444P (25%) T369M (25%)	American	Clark *et al*. 2007
88	102	5.68	0.98	Genotyping for N370S, L444P, IVS2≦1 K198T, R329C, 84insGG, and RecNciI	L444P, N370S, and RecNciI	RecNciI (60%)	Canadian	Sato *et al*. 2005

## *GBA1*‐associated PD (PD‐*GBA1*) – clinical and biochemical presentation

Individual PD patients with *GBA1* mutations cannot be distinguished at the clinical level from idiopathic PD patients without *GBA1* mutations. Parkinson‐*GBA1* (PD‐*GBA1*) patients exhibit the classic triad of bradykinesia, rigidity, and tremor, with asymmetric onset (Goker‐Alpan *et al*. 2008). However, age of onset tends to be slightly younger, an incidence of neuropsychiatric features (such as depression, anxiety, hallucination, and sleep disturbance) is higher, and there is a greater risk for earlier and more prevalent cognitive impairment in PD‐*GBA1* patients (Tan *et al*. 2007; Neumann *et al*. 2009; Sidransky *et al*. 2009; Brockmann *et al*. 2011; McNeill *et al*. 2012b; Winder‐Rhodes *et al*. 2013). The pattern of cognitive dysfunction in *GBA1*‐positive carriers is subtly different, present in those even without PD at the time of investigation (Zokaei *et al*. 2014). Nigrostriatal imaging with fluorodopa positron emission tomography or single photon emission tomography with dopamine sensitive ligands in PD‐*GBA1* demonstrate an asymmetric pattern of abnormality indistinguishable from idiopathic PD (Goker‐Alpan *et al*. 2012; McNeill *et al*. 2013b). This contrasts with the imaging of parkin (parkin RBR E3 ubiquitin protein ligase, *PARK2*) or phosphatase and tensin homolog‐induced putative kinase 1 (*PINK1*) mutant PD, where abnormalities are usually symmetrical. Patients with *GBA1* mutations also exhibit retinal thinning as determined by optical coherence tomography compared to matched controls and similar to that seen in PD patients (McNeill *et al*. 2013a).

Current evidence suggests that *GBA1* mutant homozygote and heterozygote carriers without clinical evidence of PD, exhibit the prodromal features of the disease. Olfactory function and cognitive assessment were significantly reduced, and motor testing abnormal in *GBA1*‐positive cases without features of PD compared to controls (McNeill *et al*. 2012b). A 2 year follow‐up showed significant deterioration in scores for depression, rapid eye movement sleep behavior disorder, cognition, olfaction, and motor scores (Beavan *et al*. 2015). The *GBA1* cohort exhibits a relatively rapid evolution of non‐motor and motor features.

The response to dopaminergic therapy in PD‐*GBA1* appears to be the same as that seen in idiopathic PD (Ziegler *et al*. 2007)., including the development of motor complications. In one center, retrospective genetic analysis identified *GBA1* mutations in 17% of those who had undergone deep‐brain stimulation, and in whom clinical effect was as good as those without mutations (Angeli *et al*. 2013).

Importantly, the pathology of PD‐*GBA1* is identical to that of idiopathic PD with nigral dopamine loss and Lewy bodies and neurites containing alpha‐synuclein (Westbroek *et al*. 2011; Swan and Saunders‐Pullman 2013).

## Pathogenesis of PD‐*GBA1*

The mechanism by which *GBA1* mutations increase the risk of PD is still unknown. However, given that clinical manifestation and pathological findings are almost identical in PD‐*GBA1* and idiopathic PD patients, it is thought that, as in idiopathic PD, alpha‐synuclein accumulation, mitochondrial impairment, autophagic dysfunction, inflammation, and oxidative and endoplasmic reticulum stress may play an important role in both the development and progression of PD‐*GBA1* (Schapira and Tolosa 2010).

In contrast to autosomal recessive forms of PD (caused by homozygous mutations in *PARK2*,* PINK1*, or *DJ‐1*) and autosomal dominant forms of PD (caused by heterozygous mutations in alpha‐synuclein (*SNCA*) or leucine‐rich repeat kinase 2), PD‐*GBA1* supports both autosomal recessive and autosomal dominant modes of inheritance (Sidransky 2012).

Autosomal recessive inheritance is generally associated with loss‐of‐function of mutated proteins, and several characteristics of *GBA1* suggest loss‐of‐function of mutated GCase.

- A subset of PD‐*GBA1* patients is identified with null *GBA1* alleles, such as 84GG and IVS2+1G>A, which do not encode GCase (Aharon‐Peretz *et al*. 2004; Gan‐Or *et al*. 2008; Clark *et al*. 2009).
- Inhibition of *GBA1* with condruitol β‐epoxide (CBE) in cell and animal models leads to alpha‐synuclein accumulation (Manning‐Boğ *et al*. 2009; Cleeter *et al*. 2013).
- Homozygous knock‐out of the *Gba1* gene in mice leads to mitochondrial dysfunction, and lipid and alpha‐synuclein accumulation (Enquist *et al*. 2007; Osellame *et al*. 2013).

Autosomal dominant inheritance is often associated with gain‐of‐function of mutated proteins, and several features of *GBA1* suggest gain‐of‐function of mutated GCase.

- Most PD‐*GBA1* patients are identified with heterozygous *GBA1* mutations.
- The missense mutations resulting in production of misfolded GCase account for the majority of *GBA1* mutations.
- Misfolded GCase interacts directly with alpha‐synuclein, which subsequently leads to increased alpha‐synuclein accumulation (Sidransky and Lopez 2012).

Even though there is evidence supporting loss‐ and gain‐of‐function of GCase, both hypotheses have limitations, as presence of null mutations in some PD‐*GBA1* patients contradicts the gain‐of‐function theory, while development of PD‐*GBA1* in individuals carrying heterozygous *GBA1* mutations contradicts the loss‐of‐function theory. Furthermore, misfolded GCase and loss‐of‐function may result in a secondary gain‐of‐function‐type effect through ER trapping and ER‐associated degradation process (ERAD). Even more importantly, neither the loss‐ nor gain‐of‐function hypotheses explain why the majority of individuals with *GBA1* mutations do not develop PD.

## GCase and alpha‐synuclein

PD‐*GBA1* belongs to a group of diseases collectively known as synucleinopathies, which are characterized by the presence of Lewy bodies and neurites containing SNCA. The importance of SNCA in the pathology of PD‐*GBA1* prompted a question about the GCase and SNCA relationship. To date, three main hypotheses linking GCase with alpha‐synuclein have been suggested.

The first proposes that a gain‐of‐function by the misfolded GCase results in its direct interaction with alpha‐synuclein, which then leads to increased SNCA accumulation and aggregation (Sidransky and Lopez 2012). This would require that GCase is able to interact directly with SNCA, and that GCase is present in abnormal protein aggregates containing SNCA, such as Lewy bodies. Indeed, direct interaction between GCase and the C‐terminus of SNCA was shown to occur in lysosomes (Yap *et al*. 2011). The presence of GCase in the brain tissues samples from PD‐*GBA1* patients was detected in 32–90% of Lewy bodies and neurites, showing that mutant GCase and SNCA co‐localize *in vivo* (Goker‐Alpan *et al*. 2010). One piece of evidence supporting a gain‐of‐function by the misfolded GCase was provided by a cell model study, where over‐expression of mutant GCase (containing N370S, L444P, D409H, D409V, E235A, or E340A) in neural MES23.6 and pheochromocytoma (PC12) cells led to a 21 to 148% increase in SNCA levels (Cullen *et al*. 2011). The relationship between misfolded GCase and SNCA was further strengthened by animal model studies. Namely, a progressive increase in SNCA levels was shown in the hippocampus of a homozygous D409V *Gba1* mouse model, and a significant increase in SNCA levels was detected in the forebrain and cerebellum of hypomorphic prosaposin mice carrying the homozygous V394L *Gba1* mutation (Cullen *et al*. 2011; Sardi *et al*. 2013). Also, a progressive increase in SNCA levels, from low levels to substantial SNCA aggregates, was observed in the cortex of hypomorphic prosaposin mice carrying the homozygous D409H *Gba1* mutation (Xu *et al*. 2014).

The second hypothesis proposes that a loss‐of‐function of GCase (GCase deficiency because of, for example, degradation of the misfolded enzyme) leads to accumulation of a substrate (glucocerebroside) that in turn perturbs lipid homeostasis and subsequently affects alpha‐synuclein trafficking, processing, and clearance. This eventually promotes alpha‐synuclein aggregation and facilitates alpha‐synuclein oligomer formation (Mazzulli *et al*. 2011; Westbroek *et al*. 2011; Sidransky and Lopez 2012). Further evidence supporting this hypothesis and its consequences on SNCA accumulation was provided from cell and animal model studies examining the effect of *GBA1* inhibition by CBE (Manning‐Boğ *et al*. 2009; Cleeter *et al*. 2013). Namely, treatment of differentiated SH‐SY5Y cells with CBE resulted in increased levels of SNCA (Manning‐Boğ *et al*. 2009; Cleeter *et al*. 2013). Single injection of CBE led to about 20% increase in SNCA levels in the ventral mesencephalon of normal mice. Also, enhanced SNCA immunoreactivity was observed within the cell bodies of the substantia nigra pars compacta and within the cytoplasm and cell nuclei of A9 neurons of CBE‐treated mice (Manning‐Boğ *et al*. 2009). Finally, SNCA accumulation and SNCA oligomer formation was shown in a mouse model carrying homozygous knock‐out of *Gba1*, i.e. in mice modeling loss‐of‐function of GCase (Osellame *et al*. 2013).

The third hypothesis proposes the existence of a bidirectional feedback loop in which GCase deficiency facilitates formation of alpha‐synuclein oligomers, with the subsequent increase in alpha‐synuclein oligomers leading to further decrease in normal GCase activity, which in turn promotes formation of additional alpha‐synuclein oligomers (Mazzulli *et al*. 2011). It has also been demonstrated in cell models that increased alpha‐synuclein causes a decrease in GCase activity and protein levels, as over‐expression of exogenous SNCA in SH‐SY5Y cell lines resulted in about 44–70% decrease in GCase activity, and about 33–87% decrease in GCase protein levels (Gegg *et al*. 2012).

Finally, although a majority of the studies conducted to date provide a link between GCase and SNCA via loss‐, gain‐of function, or bidirectional feedback loop, some studies failed to support the relationship between SNCA and mutant GCase. The first such study showed that *GBA1* inhibition by CBE in both differentiated SH‐SY5Y cells and rat cortical neuronal cultures did not significantly increase SNCA accumulation (Dermentzaki *et al*. 2013). This contradicts the results obtained by others, which showed an increase in SNCA levels in CBE‐treated differentiated SH‐SY5Y cells (Manning‐Boğ *et al*. 2009; Cleeter *et al*. 2013). This difference may simply have been due to the shorter exposure in the Dermentzaki study. The second such study demonstrated that *GBA1* inhibition by CBE in PC12 cell line did not alter SNCA levels, and that over‐expression of human wild‐type *GBA1* did not lead to an increase in SNCA levels in neural MES23.5 cell line. The same study, however, showed that over‐expression of wild‐type *GBA1* in HEK293‐SNCA [A53T] and PC12 cell lines resulted in decrease of SNCA levels, and that over‐expression of different mutant GCase in MES23.6 and PC12 cells led to a significant increase in SNCA levels (Cullen *et al*. 2011). The cell model specificity might explain the observed discrepancy in the effect of over‐expression of wild‐type *GBA1* on SNCA levels.

Altogether, although convincing evidence supporting the relationship between SNCA and both gain‐ and loss‐of‐function of mutant GCase exists, neither of them explains why only a proportion of individuals with *GBA1* mutations develop PD. One plausible explanation might be that in order to develop PD‐*GBA1*, in addition to the *GBA1* mutation, there must be additional genetic alternations. Another credible explanation might be that mutated GCase on its own is not sufficient to induce alpha‐synuclein pathology: perhaps only when other changes occur (e.g. a perturbation of a component of the lysosomal degradation pathway resulting in defective SNCA clearance) can PD‐*GBA1* develop.

## GCase and mitochondria

Mitochondria not only play a central role in energy production by oxidative phosphorylation but are also involved in many other cellular processes, such as synthesis of steroids and regulation of calcium homeostasis, membrane potential, apoptosis, and stress response. Taking into account the plethora of mitochondrial functions, perhaps not surprisingly, mitochondrial impairment plays an important role in the pathogenesis of PD (Schapira *et al*. 1989, 1990). In neurons, mechanistically, accumulation of dysfunctional mitochondria results in generation of reactive oxygen species and free radicals leading eventually to neuronal death. Mutations in *PARK2*,* PINK1*, and Parkinson protein 7 (*PARK7* or *DJ‐1*) genes, which affect mitochondrial morphology and function, have been identified as causing familial PD (Schapira 2008). However, the role of mitochondrial dysfunction in the pathogenesis of PD‐*GBA1* remains elusive and, to date, only three studies have investigated the impact of *GBA1* mutations on mitochondrial function. The first study showed that inhibition of *GBA1* by CBE in SHSY‐5Y cells led to a decrease in adenosine diphosphate phosphorylation, decline in mitochondrial membrane potential, and increase in free radical generation, so demonstrating that GCase loss‐of‐function causes oxidative stress and mitochondrial dysfunction (Cleeter *et al*. 2013). The second study showed that loss‐of‐function of GCase in a mouse model carrying homozygous knock‐out of *GBA1* resulted in accumulation of dysfunctional and fragmented mitochondria, and reduction in respiratory chain complex activities, membrane potential, and oxygen consumption (Osellame *et al*. 2013). The third study showed that gain‐of‐function of GCase in hypomorphic prosaposin mice carrying homozygous D409H or V394L *Gba1* mutation led to significant reduction in oxygen consumption and mitochondrial adenosine triphosphate (ATP) production. The same findings were also observed in wild‐type cerebral cortical neural cells treated with CBE (Xu *et al*. 2014). Altogether, data from cell and animal model studies demonstrated that both loss‐ and gain‐of‐function *GBA1* mutations cause mitochondrial impairment, and so it would be worth investigating whether mitochondrial dysfunction is also present in PD‐*GBA1* brains.

## GCase and autophagy

Autophagy is a lysosomal pathway that is involved in degradation of damaged organelles, such as mitochondria and endoplasmic reticulum, and clearance of long‐lived misfolded or aggregated proteins, such as alpha‐synuclein. Three distinct types of autophagy have been identified: macroautophagy, microautophagy, and chaperone‐mediated autophagy (CMA). Increasing evidence indicates the existence of a strong link between impairment of autophagy and PD. Dysfunction of autophagy has been shown both in brain tissue samples from idiopathic PD patients and toxic mouse models of PD (Chu *et al*. 2009; Alvarez‐Erviti *et al*. 2010; Dehay *et al*. 2010; Vila *et al*. 2011). Although involvement of autophagic impairment in the development of GD and especially PD is not yet fully understood, emerging data clearly suggest its importance.

Lysosomal dysfunction resulting in progressive accu‐mulation of glucocerebroside plays a central role in GD pathogenesis. Accumulation of sphingolipids, to which glucocerebroside belongs, has been shown to alter autophagy by both inducing cell death and reducing autophagosome clearance, and so promoting their accumulation (Tamboli *et al*. 2011). Indeed, induction of autophagy has been demonstrated in GD human fibroblasts that showed accumulation of glucocerebroside, while increased number of autophagosomes has been shown in hypomorphic prosaposin mice carrying the homozygous V394L *Gba1* mutation that showed accumulation of glucocerebroside (Sun *et al*. 2010a,b; Vaccaro *et al*. 2010). However, it seems unlikely that the autophagic dysfunction in PD‐*GBA1* is because of accumulation of glucocerebroside (and glucosphingosine, a deacetylated glucocerebroside), since substrate accumulation is believed to require both *GBA1* alleles to carry a genetic alteration, and this is not the case in the majority of PD‐*GBA1* individuals. Analysis of putamen and cerebellum samples from PD‐*GBA1* patients supports that notion, as no accumulation of glucocerebroside and glucosphingosine was observed in these brain regions (Gegg *et al*. 2015). In contrast, significantly increased glucosphingosine levels (and significantly reduced GCase activity) were detected in the substantia nigra and hippocampus of idiopathic PD patients during sixth and seventh decade of life, respectively (Rocha *et al*. 2015). However, no changes in glucosphingosine levels were observed in the putamen and cerebellum of idiopathic PD patients, although a significant reduction in GCase activity was observed in these regions (Gegg *et al*. 2015; Rocha *et al*. 2015). The observed discrepancy in glucosphingosine accumulation among different brain regions should prompt further analysis in both of PD‐*GBA1* and idiopathic PD patients to establish whether the relationship between reduction of GCase activity and accumulation of glucosphingosine exists, and, if indeed there is such a link whether autophagic dysfunction should be at least partially attributed to GCase substrate accumulation.

The relationship between SNCA and both gain‐ and loss‐of‐function of mutant GCase provides a plausible explanation linking autophagic impairment with PD‐*GBA1*. It has been shown that SNCA is preferentially degraded by CMA, and that impairment of CMA leads to accumulation and aggregation of SNCA in SH‐SY5Y cell lines (Alvarez‐Erviti *et al*. 2010). Conversely, it has been demonstrated that expression of mutant SNCA leads to CMA impairment in proliferating PC12 and SH‐SY5Y cells, while over‐expression of wild‐type SNCA results in CMA dysfunction in differentiated SH‐SY5Y cells (Xilouri *et al*. 2009). The latter observation is especially interesting, as misfolded GCase has been shown to facilitate SNCA accumulation and aggregation (Mazzulli *et al*. 2011; Westbroek *et al*. 2011; Sidransky and Lopez 2012), and so it can be speculated that SNCA increase because of GCase dysfunction might lead to CMA impairment. Thus, finding a therapeutic agent that is able to increase levels of functional, properly folded GCase would enhance SNCA degradation by CMA, and consequently lead to reduction in SNCA levels.

## GCase and endoplasmic reticulum stress

Secretory and membrane‐associated proteins are synthetized in the endoplasmic reticulum and newly synthetized proteins are folded in the ER with the help of ER chaperones. Proteins that fail to fold correctly are recognized by the ER quality control system and retained for refolding. Misfolded proteins that fail to refold by ER chaperones are disposed of by ERAD via the ubiquitin–proteasome system. When the number of misfolded proteins exceeds the capacity of the ERAD system, misfolded proteins accumulate in the ER causing stress. In response to such stress, the unfolded protein response (UPR) is activated to help restore normal cell function. If this fails, and ER stress continues, malfunctioning cells are eliminated by apoptosis (Yoshida 2007). Emerging evidence shows the involvement of ER stress in the pathogenesis of PD (Imai *et al*. 2001; Ryu *et al*. 2002). The direct link between ER stress and PD comes from studies of parkin (PARK2), which in mutated form causes early onset of PD. Parkin, an E3 ubiquitin ligase is a member of the UPR, where it is responsible for ubiquitination of misfolded proteins for degradation. Clearance of misfolded proteins is impaired when parkin is mutated, which eventually results in ER stress (Imai *et al*. 2000, 2001). The link between misfolded GCase and ER stress is very plausible, as prolonged accumulation of misfolded GCase in the ER would eventually cause ER stress and UPR activation. Misfolded GCases, including N370S and L444P mutants, have been shown to be retained in the ER and undergo ERAD in human GD fibroblasts (Ron and Horowitz 2005; Bendikov‐Bar *et al*. 2011). Moreover, it has been reported that GD severity shows correlation with levels of misfolded GCase retention in the ER and so, speculatively, with ER stress (Ron and Horowitz 2005). Biochemical analysis of PD‐*GBA1* putamen samples for the presence of UPR markers showed a 63% increase in C/EBP homologous protein levels and a 26% increase in binding immunoglobulin protein. The observed UPR/ERAD could be caused by misfolded GCase, but other causes such as oxidative stress, mitochondrial dysfunction, proteolysis, or altered calcium homeostasis cannot be excluded (Gegg *et al*. 2012). The direct interaction of misfolded GCase with parkin, and its subsequent ERAD degradation, has been observed in human COS7 and HEK293 cells, providing evidence for the existence of a direct link between PD‐*GBA1* and ERAD. Misfolded GCase‐parkin interaction can block parkin interaction with other protein that are degraded via parkin‐mediated ERAD, which would result in further accumulation of proteins in the ER and a further increase in ER stress that would consequently result in neuronal death, and so PD development (Ron *et al*. 2010). In contrast to human studies, analysis of UPR markers in the neuronal cultures and tissues obtained from transgenic GD mouse models and CBE‐treated normal mice showed no alternations in C/EBP homologous protein, binding immunoglobulin protein, and X‐box binding protein 1 (XBP1) (Farfel‐Becker *et al*. 2009). Further studies are required to establish whether ER stress really contributes to the pathogenesis of PD‐*GBA1*.

## PD‐*GBA1* treatment

There is no specific treatment available for PD‐*GBA1* patients to modify the course of the disease. Since nigral dopamine loss observed in individuals with PD‐*GBA1* is identical to that observed in individuals with idiopathic PD, PD‐*GBA1* patients are normally given dopaminergic therapy to alleviate the dopamine deficit in the striatum. Therefore, the development of new therapies that can slow down disease progression is urgently required. The increasing evidence linking GCase with alpha‐synuclein and mitochondrial pathways has relevance to a potential novel therapeutic strategy for both PD‐*GBA1* and idiopathic PD patients. Moreover, even patients without *GBA1* mutations show significant reduction in GCase activity in the substantia nigra (Gegg *et al*. 2012). This highlights the importance of GCase function, not only in PD‐*GBA1* but also in idiopathic PD and suggests that *GBA1* treatments may potentially prove to be effective with many, if not all, PD patients (Schapira and Gegg 2013). The use of small molecular chaperones such as *N*‐(*n*‐nonyl)deoxynojirimycin, isofagomine, pyrazolopyrimidines, or ambroxol (i.e. the same chaperones that are currently being tested for their application in treating GD) may be of interest as a novel therapy for PD, as a means to decrease alpha‐synuclein levels and improve mitochondrial function (Sawkar *et al*. 2002; Steet *et al*. 2006; Maegawa *et al*. 2009; Bendikov‐Bar *et al*. 2011, 2013; Patnaik *et al*. 2012; Luan *et al*. 2013; Sanders *et al*. 2013; McNeill *et al*. 2014). Interestingly, the benefits of increasing wild‐type GCase stability, trafficking, and activity in idiopathic PD have been demonstrated through the study of mice over‐expressing human alpha‐synuclein under the murine Thy‐1 promoter (Thy1‐aSyn). Administration of a molecular chaperone AT2101 (afegostat‐tartrate, isofagomine) led to GCase increase in the brain and resulted in improvement of both motor function and neuropathological manifestations (such as elimination of microglial inflammatory response and reduction in alpha‐synuclein immunoreactivity in the substania nigra) in Thy1‐aSyn mice (Richter *et al*. 2014). These results show that an increase in GCase activity achieved by administration of molecular chaperones might significantly improve clinical and biochemical manifestations of synucleinopathies, even those without *GBA1* mutations, and so further development of small molecular chaperones gives a great promise for finding a successful treatment not only for PD‐*GBA1*, but also for idiopathic PD and other synucleinopathies.

## Conclusions

After Alzheimer, Parkinson disease is the second most common neurodegenerative disorder. The most common risk factor for PD involves mutations in the *GBA1* gene, which occurs in 5–10% of PD patients (Sidransky *et al*. 2009; Bultron *et al*. 2010; McNeill *et al*. 2012a). This compares with leucine‐rich repeat kinase 2 mutations that are estimated to cause 0.5% of sporadic PD. Further, even in patients without *GBA1* mutations, a decrease in GCase activity is still found in all studied cases, suggesting that this protein plays a much more important role in PD than previously appreciated. As such, further studies of *GBA1*, in particular of treatments that increase levels of GCase, are likely to be one of the most promising future avenues for tackling PD.
